# Potential Pathogenicity of *Candida* Species Isolated from Oral Cavity of Patients with Diabetes Mellitus

**DOI:** 10.1155/2021/9982744

**Published:** 2021-05-26

**Authors:** Hasti Nouraei, Mehdi Ghaderian Jahromi, Leila Razeghian Jahromi, Kamiar Zomorodian, Keyvan Pakshir

**Affiliations:** ^1^Department of Parasitology and Mycology, School of Medicine, Shiraz University of Medical Sciences, Shiraz, Iran; ^2^Department of Radiology, School of Medicine, Medical Imaging Research Center, Shiraz University of Medicine Sciences, Shiraz, Iran; ^3^Department of Psychiatry, School of Medicine, Shiraz University of Medicine Sciences, Shiraz, Iran; ^4^Basic Sciences in Infectious Diseases Research Center, School of Medicine, Shiraz University of Medical Sciences, Shiraz, Iran

## Abstract

**Introduction:**

In the recent decade, the increased immunocompromised population such as diabetic patients makes a high incidence of invasive *Candida* infections. Diabetes mellitus is the most common endocrine metabolic disorder, and diabetic patients are more susceptible to oral candidiasis infection. Candidiasis is an opportunistic fungal infection caused by many species of *Candida*. Secretion of exoenzymes plays an important role in the virulence and pathogenesis of *Candida* species. The aim of this study was to evaluate the potential role of phospholipase, esterase, and hemolytic activity of *Candida* species isolated from oral cavity lesions of diabetic patients.

**Methods:**

A total of 108 *Candida* species including 75 *Candida albicans* and 33 non-*Candida albicans* species were recovered from the oral cavity of diabetic patients included in our study. Egg yolk agar, Tween 80 opacity medium, and blood agar plate assays were used for determining phospholipase, esterase, and hemolytic activities, respectively.

**Results:**

*Candida albicans* species had the most exoenzyme activity in comparison to non*-albicans* isolates. *Candida albicans* isolates showed 97.3%, 100%, and 77.3% phospholipase, hemolysin, and esterase activities, respectively. The difference between *Candida albicans* and non-*Candida albicans* was significant in phospholipase (*P* < 0.001) and hemolytic activity (*P* = 0.027), but not significant in esterase activity (*P* = 0.076).

**Conclusion:**

This study showed that most of the isolates had different enzymatic patterns, and *Candida albicans* isolates had the most exoenzyme activity. So due to the potential effects of these enzymes in pathogenesis and virulence effects of *Candida* species, we can conclude that the severity of extracellular enzymes may play a role in the severity of signs and symptoms of *Candida* oral cavity infections in diabetic patients.

## 1. Introduction

Diabetes mellitus (DM) is one of the most prevalent metabolic chronic disorders throughout the world caused by the dysfunction of *β* cells in pancreatic islet 1 in which glucose plasma levels remain high for a long period [1, 2]. Among many reasons that caused the high morbidity of these diseases, chronic macro- and microvascular complications such as neuropathy, nephropathy, retinopathy, and heart complications are more important [3]. The rates of this disease are equal in both genders and affect more than 425 million people all around the world [4]. In the 21st century, DM is becoming a global epidemic and one of the largest emerging threats to public health which significantly affect the quality of life and longevity of the patients, as well as healthcare costs [5, 6].

The overall prevalence of diabetes among adults over 18 years old has increased during the past decades, and the World Health Organization (WHO) predicts that this will increase to 439 million, almost 10% of adults in 2030 [7]. Diabetes mellitus is usually associated with systemic complications, such as kidney disease, eye disease, recurrent fungal skin infection, and oral diseases, including gingivitis, periodontitis, and candidiasis [2, 8].

Diabetes mellitus is divided into two types: insulin-dependent (type 1) and non-insulin-dependent (type 2). Ninety percent of people have type 2 diabetes mellitus (DM2) that mainly has correlation with personal lifestyle, including high-calorie diets, low physical activity, and smoking [9].

Diabetes mellitus has many oral manifestations, complication, and infections such as dry mouth, burning mouth syndrome (BMS), taste disorders, oral candidiasis, rhinocerebral zygomycosis (mucormycosis), aspergillosis, geographic tongue, oral lichen planus, delayed wound healing, periodontal disease, gingivitis, and increased incidence of infection [10].

Yeast infections in diabetic patients are common [11]. *Candida* spp. is frequently found in patients with poor glycemic control, and it was already shown that increased oral *Candida* carriage is related to increasing levels of glucose in saliva [12]. Oral candidiasis is commonly caused by *C*. *albicans* species and presents many clinical forms (pseudomembranous candidiasis, cheilitis, and stomatitis) [13].

Some factors have a major influence on the balance between host and yeasts and caused the transition of *Candida* sp. (*C. albicans*, *C. glabrata*, *C. tropicalis*, *C. parapsilosis*, and *C. krusei*) [14] from commensal to pathogen and causing infection such as reduced salivary flow, higher salivary glucose levels, and impaired candidacidal activity of neutrophils [15]. The risk factors for oral candidiasis are complex, but it is known that tongue lesions, tobacco smoking, alcohol consumption, denture wearing, the intake of medication, and immunosuppression (e.g., diabetes mellitus) clearly influence oral candidiasis [16]. However, in patients with DM that are immunocompromised, these species can cause invasive infections. Several studies have shown the link between *Candida* sp. infection and DM [17]. Higher expressions in enzymatic activity and ability of biofilm formation in *Candida* spp. are two of the most important features in oral candidiasis [18].

Many studies have established a relationship between hydrolytic enzyme activity and an increase in the pathogenic ability of *Candida* spp. [6, 19].

Due to higher blood glucose concentration in diabetic patients, *Candida* spp. isolates present significantly higher hemolytic, esterase, and phospholipase enzymatic activity [20, 21]. Another hypothesis was that these species are more pathogenic in DM because of abnormal conditions [22]. They also can cause inflammatory reactions by increasing vascular permeability and clinical symptoms, which may disturb the humoral host defense [23]. These enzymes could digest and damage the membrane, initiating cell lysis and facilitating the penetration of the infecting fungi [24].

So, due to this fact that the presence of high enzymatic activity in *Candida* species oral candidiasis could exacerbate different clinical symptoms in DM patients, we decided to design this study to evaluate this potential pathogenicity of *Candida* species isolated from the oral cavity of patients with diabetes mellitus.

## 2. Materials and Methods

### 2.1. Yeast Isolates

In this study, a total of 108 stock *Candida* species consisting of 75 *Candida albicans* and 33 non-*albicans Candida* (NAC) species including 16 *C. dubliniensis*, 9 *C. glabrata*, 3 *C. parapsilosis*, 2 *C. guilliermondii*, 1 *C. tropicalis*, 1 *C. krusei*, and 1 *C. kefyr* which previously were recovered from diabetic patients were used. These species were identified to the species level based on results of conventional methods such as the ability to produce germ tubes, chlamydoconidia production in corn meal agar medium, and colony color on chromogenic media (CHROMagar *Candida*) and molecular method (PCR-RFLP) [25].

### 2.2. Determination of Phospholipase Activity

*Candida* species isolates were screened for phospholipase activity by measuring the size of the zone of precipitation after growth on egg yolk agar [26]. The egg yolk medium consisted of 58.4 g NaCl and 5.5 g CaCl_2_ and 65 g Sabouraud dextrose agar and 2% sterile egg yolk.

First, the components without the egg yolk were mixed and sterilized; then, the egg yolk was centrifuged at 5,000 × g for 30 min, and 20 ml of the supernatant was added to the sterilized medium. The inocula were prepared (McFarland 2 turbidity) and were deposited onto the egg yolk agar medium and left to dry at room temperature. Each culture was then incubated at 37°C for 10 days, after which the diameter of the precipitation zone (Pz) was determined. Phospholipase activity was expressed as the ratio of the diameter of the colony to the diameter of the colony plus the precipitation zone (in mm) [26].

### 2.3. Determination of Esterase Activity

Esterase activity was measured using the Tween 80 opacity test medium [27], which was prepared with 10 g of bacteriological peptone (Merck, Germany), 5 g of sodium chloride, 0.1 g of calcium chloride, 15 g of agar, and 1000 ml of distilled water. After, the medium was autoclaved; then, 5 ml of autoclaved Tween 80 was added (Merck, Germany). Then, 10 microlitres of *Candida* suspension (10^6^ cells/ml) was inoculated as a spot and was incubated at 30°C for 10 days. The colony diameter (*a*) and the diameter of colony plus precipitation zone (*b*) were measured. The esterase activities were expressed as Ez value (*a*/*b*) as described by Price et al. [26].

### 2.4. Determination of Hemolysin Activity

Hemolytic activity was evaluated with a blood plate assay [28]. Sheep blood SDA were prepared by adding 7 ml of fresh sheep blood to 100 ml of SDA supplemented with 3% glucose. Suspensions equal to McFarland 2 turbidity from the pure culture of the yeast colonies were prepared. Ten microliters of this suspension was spotted on prepared media and incubated at 37°C for 48 h. For hemolytic activity, a ring of lysis around the colonies was considered complete (in case of a totally translucent ring; beta), incomplete (in case of greenish-black halo; alpha), and no hemolysis (gamma or none). *Candida albicans* ATCC10261 was used as a control for all three methods.

### 2.5. Statistical Analysis

Results were analyzed using the SPSS22 (Statistical Package for the Social Sciences) program. The Fisher exact test was considered for statistical significance with a *P* value less than 0.05.

### 2.6. Ethical Approval

This project was found to be in accordance with the ethical principles and the national norms and standards for conducting medical research in Iran and has been approved by the research ethics committee (IR. SUMS.REC.1390.3755).

## 3. Results

### 3.1. Phospholipase Activities of *Candida* spp.

In total, out of 75 samples of *Candida albicans*, 73 (97.3%) isolates had phospholipase activity, and 69 species (92%) showed +4 positive for phospholipase activity and only 2 isolates (2.7%) had no phospholipase activity. However, out of 33 non-*Candida albicans* samples, only 20 (60.6%) isolates had +4 positive phospholipase activity and 11 (33.3%) isolates had no phospholipase activity. Among non-*Candida albicans* samples, all of the *C. dubliniensis* isolates showed +4 positive phospholipase activities (100%), but *C. krusei* and *C. guilliermondii* had no phospholipase activity. More details are explained in Table 1. There were statistically significant differences in phospholipase activity between two groups of *C. albicans* and non-*albicans* (*P* < 0.001).

### 3.2. Esterase Activities of *Candida* spp.

Out of 75 samples of *C. albicans*, 58 (77.3%) isolates had esterase activity, and 52 isolates (69.3%) showed +4 positive esterase activity. On the other hand, 17 isolates (22.7%) had no esterase activity. Among non-*Candida albicans* species, 21 (63.6%) isolates had esterase activity and 17 (51.51%) isolates showed +4 positive esterase activities (Table 1). There was no significant statistical correlation between the two groups of *Candida albicans* and non-albicans species in esterase activities (*P* = 0.07).

### 3.3. Hemolytic Activities of *Candida* spp.

**Figure 1:**
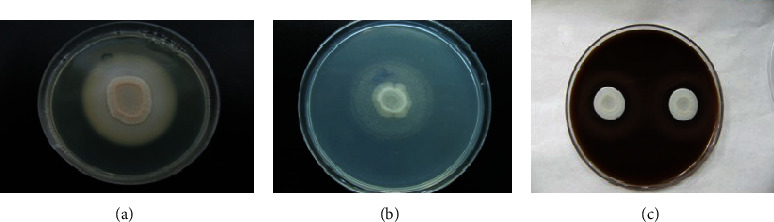
(a) Phospholipase production of *Candida spp.* isolates on egg yolk agar. (b) Positive activity of esterase in *Candida* species. (c) Showed beta-hemolytic activity.

All of the 75 studied samples of *C. albicans* (100%) had beta-hemolytic activity. Out of the 33 samples of non-*Candida albicans*, 30 isolates (91%) had beta-hemolytic activity and 3 isolates (9%) had alpha-hemolytic activity that included 1 *C. dubliniensis* and 2 *C. glabrata*. None of the isolates had gamma-hemolytic activity. Statistical analysis for production of hemolysin between *C. albicans* species and non-*Candida albicans* species showed that there was a significant difference between 2 species (*P* = 0.027) (Figure 1).

## 4. Discussion

Diabetes mellitus characterized by hyperglycemia resulting from reducing insulin secretion, insulin action, or both is an endocrine disease that is a global health problem [29]. An increasing level of glucose in serum causes variation damage to different types of cells, such as endothelial cells, neurons, renal cells, and keratinocytes [30]. An increasing glucose level in serum could damage monocyte and neutrophil adherence, chemotaxis, and phagocytosis which are important in host defense. During *Candida* infection, raising glucose in the cell causes increasing adherence and invasion of organisms to the infected tissues [31]. Oral candidiasis can be diagnosed by the differential patterns of mucosal changes like erythematous, pseudomembranous, and curd-like plaques [32].

A previous study showed increases in the prevalence of *C. albicans* strains in the oral cavity of patients with DM. In another study, this prevalence rate of the *Candida* species was 87.5% [33]. Fatahinia et al. demonstrated that the prevalence of *C. albicans* isolates in the oral cavity of diabetic patients was significantly greater than that of nondiabetic patients (66.7% and 57.1%), and also, they reported that there were some differences in the pattern of phospholipase, esterase, and hemolysin secretion between these 2 groups. For example, hemolytic activity was detected in 100% of isolates in the diabetic group, but this rate was 52% of isolates in the nondiabetic patient groups [21].

Many factors in *Candida* spp have been attributed to increasing the incidence of candidiasis in diabetic patients such as enzymatic activity, biofilm formation, hydrophobicity, phenotypic switching, and yeast-to-hypha transition [24]. Producing extracellular enzymes by *Candida* spp can play an important role in its pathogenicity, invasion, and destroying host tissues and effect on developing clinical signs. *Candida* spp also used their phospholipase and esterase enzymes for the invasion to host tissue and hemolysin for lysing the blood cell. Although *Candida* has the ability to produce these enzymes, the amount and strength of these enzymes are various among different species, and because of the different sources of their isolation, they have different patterns of secretion [34].

Phospholipase secretions also help the organism to penetrate better to host tissue [24]. In the present study, phospholipase activity was detected in 73 (97.3%) out of the 75 *C. albicans* isolates and in 22 (66.6%) of NCA. These results demonstrate that the group of *C. albicans* isolates produce high phospholipase enzyme which is very important for damaging tissue. Tsang et al. [20] reported that 100% of *C. albicans*-tested isolates showed phospholipase activity that their result was close to our result. This enzyme increases the ability of organisms for the destruction of immune factors of host cells and makes better opportunity for invasion and achieving nutrients [35]. Esterase activity is another virulence factor in *Candida* spp which digests the surface membrane of the host cell and causes better binding, penetration, and invasion, thus playing important roles in *Candida* pathogenicity [21].

In this study, 77.3% of *C. albicans* isolates and 63.5% of NCA showed esterase activity which was almost close between the two groups while Fatahinia et al. [21] reported that only 21.6% of their *C. albicans* isolates presented this activity, and this data was not in agreement with our result. The amount of esterase secretion could be considered an important factor in tissue invasion for organisms, and it could exacerbate symptoms in diabetic patients [36].

The other virulence factor that is important in the pathogenicity of *Candida* spp is the hemolysin enzyme that facilitates the pathogen to extract iron from molecules with hemoglobin or hemin [24]. In our study, 100% *C. albicans* of isolates had hemolytic activity; our data was in agreement with Fatahinia et al. [21] results but not in agreement with Tsang et al. [20] study by which the hemolytic activity was 100% and 80% of isolates, respectively. The presence of white and secreted plaque lesions in oral candidiasis (thrush) and also serous fluid in lesions and cheilitis (perleche) may link with the number of blood cells in the mouth and covered oral mucosa so this condition can make facility in *Candida* species hemolysin secretion and obtain iron from the blood.

Luo et al. believed that increased hemolysin activity among *Candida* spp isolates in DM patients was because of increasing blood glucose concentration, and also, an increased salivary glucose concentration in DM patients may contribute directly or indirectly to rising hemolysin production in *Candida* spp. [37]. This is another reason for the importance of this enzyme.

According to this fact that one of the important pathogenic factors in *Candida* species is their extracellular enzymes and due to the weakness of the defense barrier in diabetic patients, it can be guessed that the production of these enzymes plays an important role in severity of invasion to the mucosal cell. So, among *Candida* species isolated from oral lesions, if the production of these enzymes is more intense, the severity and extent of the lesion will probably be higher.

Here, due to the high production of enzymes in *Candida albicans* isolates, it can be inferred that the incidence of oral mucosal complications with these species is probably higher than other species and more enzyme production by these species may be one of the factors that cause these species to be more isolated from oral mucosal lesions.

## 5. Conclusion

Diabetes is a chronic health condition, and diabetic patients are more susceptible to oral candidiasis. Virulence factors like phospholipase, esterase, and hemolysin activity play an important role in the pathogenicity of *Candida* species. Knowledge about these virulence factors can be helpful in better management of these largely opportunistic fungal infections and treatment of disease. Further research aimed at characterizing the other risk factors of *Candida* species is needed for better management, preventive therapy, and treatment of diabetes mellitus.

## Figures and Tables

**Table 1:** Comparisons and distribution of virulence factors of clinical *Candida* species isolated from diabetic patients.

Species (*n*)	Phospholipase					Esterase				
	Neg	1+	2+	3+	4+	Neg	1+	2+	3+	4+
*C. albicans* (75)	2 (2.7%)	0 (0%)	1 (1.3%)	3 (4%)	69 (92%)	17 (22.6%)	0 (0%)	1 (1.3%)	5 (6.7%)	52 (69.4%)
*Non-Candida albicans* (33)	11 (33.4%)	0 (0%)	0 (0%)	2 (6%)	20 (60.6%)	12 (36.5%)	0 (0%)	1 (3%)	3 (9%)	17 (51.5%)

## Data Availability

The data used to support the findings of this study were supplied by Shiraz University of Medical Sciences under license and so cannot be made freely available. Requests for access to these data should be made to Keyvan Pakshir, pakshirk@gmail.com.
